# Identification of anti-flaviviral drugs with mosquitocidal and anti-Zika virus activity in *Aedes aegypti*

**DOI:** 10.1371/journal.pntd.0007681

**Published:** 2019-08-20

**Authors:** Shengzhang Dong, Seokyoung Kang, George Dimopoulos

**Affiliations:** W. Harry Feinstone Department of Molecular Microbiology and Immunology, Bloomberg School of Public Health, Johns Hopkins University, Baltimore, Maryland, United States of America; Institut Pasteur, FRANCE

## Abstract

Zika virus (ZIKV), an emerging arbovirus belonging to the genus Flavivirus, is transmitted by *Aedes* mosquitoes. ZIKV infection can cause microcephaly of newborn babies and Guillain–Barré syndrome in adults. Because no licensed vaccine or specific antiviral treatment is available for ZIKV infection, the most commonly used approach to control the spread of ZIKV is suppression of the mosquito vector population. A novel proposed strategy to block arthropod virus (arbovirus) transmission is based on the chemical inhibition of virus infection in mosquitoes. However, only a few drugs and compounds have been tested with such properties. Here we present a comprehensive screen of 55 FDA-approved anti-flaviviral drugs for potential anti-ZIKV and mosquitocidal activity. Four drugs (auranofin, actinomycin D (Act-D), bortezomib and gemcitabine) were toxic to C6/36 cells, and two drugs (5-fluorouracil and mycophenolic acid (MPA)) significantly reduced ZIKV production in C6/36 cells at 2 μM and 0.5 μM, respectively. Three drugs (Act-D, cyclosporin A, ivermectin) exhibited a strong adulticidal activity, and six drugs (U18666A, retinoic acid p-hydroxyanilide (4-HPR), clotrimazole, bortezomib, MPA, imatinib mesylate) significantly suppressed ZIKV infection in mosquito midguts. Some of these FDA-approved drugs may have potential for use for the development of ZIKV transmission-blocking strategies.

## Author summary

Zika virus (ZIKV) is a human threat with a global health burden. As many as 86 countries and territories have reported evidence of mosquito-transmitted Zika infection, and there is no effective means of control. Recently, several studies have identified FDA-approved drugs exerting anti-ZIKV activity in mammalian cells. Here, we have screened such drugs for the ability to reduce mosquito viability or suppress ZIKV infection of mosquito cells. We identified three drugs that significantly increased mosquito mortality and six that significantly suppressed ZIKV infection in mosquito midguts. Altogether, our study provides a list of candidate agents for potential use to block ZIKV transmission in mosquitoes by chemical inhibition.

## Introduction

Zika virus (ZIKV) belongs to the Flavivirus genus, which also includes dengue virus (DENV), West Nile (WNV), yellow fever virus (YFV) and Japanese encephalitis viruses (JEV) and is mainly transmitted by *Aedes* mosquitoes, including *Ae*. *aegypti* and *Ae*. *albopictus*, in an urban cycle [1]. The typical symptoms of Zika are very similar to dengue fever, including conjunctivitis (red eyes), muscle and joint pain, mild fever, rash, and severe headache [1]. ZIKV can also be directly transmitted between humans through sexual contact, or vertically from mother to child through during pregnancy [2–4]. ZIKV was first isolated from a rhesus monkey in the Zika forest of Uganda in 1947 and was then believed to be mainly circulated in a sylvatic cycle between non-human primate hosts and mosquitoes, until an outbreak occurred in Libreville, the capital of Gabon in 2007 [5, 6]. The most recent ZIKV outbreak in the Americas gained significant public attention, since Zika infection can result in microcephaly of newborn babies and Guillain–Barré syndrome in adults [7, 8].

No approved vaccines or specific therapies to prevent or treat ZIKV infection exist. Therefore, vector population suppression remains the most effective approach to control the spread of ZIKV. Control efforts to limit the population of *Aedes* mosquitoes and prevent mosquito biting rely mainly on insecticide application, removal of mosquito breeding sites, and the use of repellents and door/window curtains. However, these control methods are plagued by limitations such as insecticide resistance and logistics that hamper disease control, and novel control strategies are therefore needed.

Following ingestion of a viremic bloodmeal from a vertebrate host, an arthropod virus (arbovirus) needs to infect and replicate in several tissues and escape the vector’s immune defenses to be transmitted to another vertebrate host during blood feeding [9–12]. Effective blocking of arbovirus infection in the mosquito vector will result in transmission blocking. Numerous mosquito-encoded virus host factors (agonists) and restriction factors (antagonists) have been identified and shown to play essential roles in influencing arbovirus infection [13]. The rather small genomes of flaviviruses, comprising about 10 genes, do not allow for much functional diversity with regard to the viruses’ interaction with the vertebrate host’s and insect vector’s cellular machineries; hence, some virus host factors are conserved between the two hosts. Accordingly, a recently explored transmission blocking strategy that is based on chemical inhibition of host factors has shown significant reduction in DENV in midgut and salivary gland after injection of, or feeding on, chemical compounds that had previously been shown to inhibit infection in vertebrate cells [14].

An ideal transmission-blocking compound should either be safe for human consumption, or be environmentally safe through delivery in attractive toxic sugar bait (ATSB) systems. In both cases the compound should either inhibit virus infection of the vector or kill the vector [15, 16]. Several studies have reported successful identification of Food and Drug Administration (FDA)-approved drugs exerting ZIKV-inhibition in mammalian cells [17–19], involving diverse mechanisms of action such as the inhibition of DNA/RNA synthesis, DNA replication, proteolysis, or purine synthesis. Such compounds could qualify for the development of the above-mentioned control strategies. Here, we report the screening of such FDA-approved drugs to evaluate their anti-ZIKV activity in mosquito cells and adult mosquitoes and their ability to influence mosquito viability.

## Results

### Anti-flaviviral candidate drugs

We searched the literature for drugs and compounds that have been reported to exert anti-ZIKV and/or anti-DENV activity in mammalian or mosquito cells, using the following criteria: 1) significant inhibition of flavivirus (ZIKV or DENV) infection in mammalian or mosquito cells; 2) no obvious toxicity to the tested cells; 3) approval already granted for human use or clinical trials. A total of 55 drugs were selected on the basis of these criteria (S1 Table). It should be noted that the anti-flaviviral activity of some drugs had been reported in multiple studies. For example, the cholesterol-lowering drug Lovastatin has been shown to inhibit ZIKV replication in the Huh7 human hepatoma cell [17, 18] as well as to have an impact on DENV RNA replication and virion secretion in primary human monocytes and cultured cell lines [20, 21].

### C6/36 cell-based anti-ZIKV drug screening

**Fig 1:**
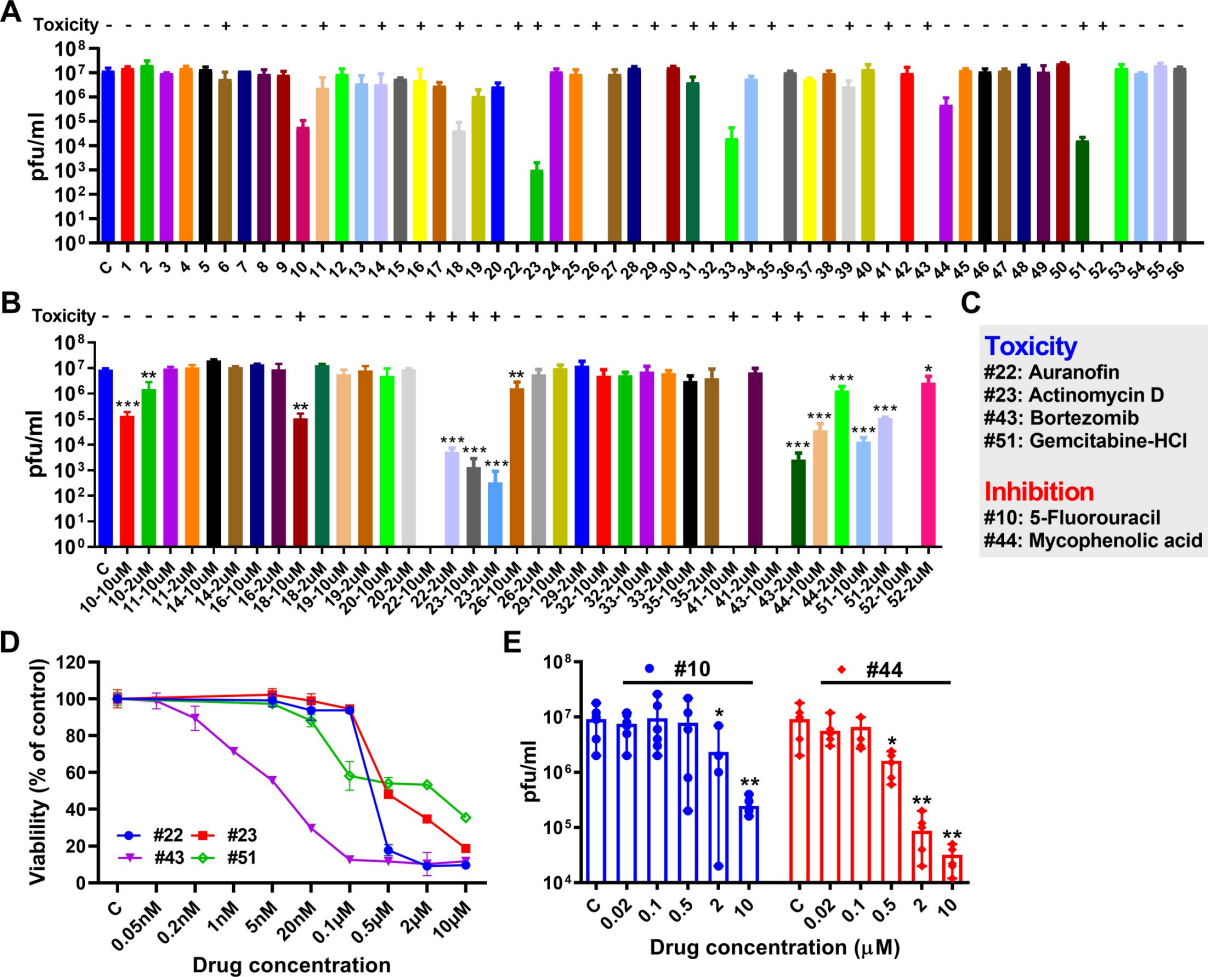
Cell toxicity and anti-ZIKV activity of the selected 55 anti-flaviviral compounds, based on testing of mosquito cells. **A**, C6/36 cells were treated for 1 h with a final concentration of 20 μM of each drug and a vehicle control (C), and ZIKV was inoculated into the cells at an MOI of 0.5. Cell mortality and virus titers were assayed at 3 days post-infection (dpi). **B**, C6/36 cells were treated with selected drugs at a final concentration of 10 μM or 2 μM. Cell mortality and virus titers were assayed at 3 dpi. **C**, List of compounds with toxicity or anti-ZIKV activity in mosquito cells. Cell mortality was assessed microscopically, looking for obvious floating and highly granulated deformed cells, and virus titers in the medium were determined by plaque assay. **D**, The effects on C6/36 viability of the identified drugs tested for different concentrations. Cell viability was measured using the CellTiter 96 One Solution Cell Proliferation Reagent. Each point represents the average ± SD percent of viable cells compared to the control (1% DMSO treated cells). **E**, Anti-ZIKV activity of the identified drugs in C6/36 cells with different concentration. Virus titers in the medium were determined by plaque assays. One replicate for A and B, three replicates for D and E were performed, and each replicate has at least three technical replicates. Columns with error bars represent the median value with range of virus titer. Significance was based on a Student’s *t*-test between the control and each drug. *P<0.05, **P<0.01, ***P<0.001.

We first used a mosquito cell line-based assay to screen the 55 selected drugs for inhibition of ZIKV infection. C6/36 cells grown overnight to 80% confluence were pre-treated for 1 h with a 20 μM concentration of each drug or the vehicle control (DMSO), and ZIKV was then inoculated into the cells at a multiplicity of infection (MOI) of 0.5. The cell mortality and virus titer in the medium were determined by microscopy and plaque assay, respectively at 3 days post-infection (dpi). We found that 15 drugs induced moderate cell mortality based on a >50% cell death as indicated by floating cells or highly granulated deformed cells, when compared to the DMSO-treated control (Fig 1A and S2 Table), and 3 drugs (5-fluorouracil, deferasirox, and mycophenolic acid [MPA]) mediated a significant reduction in virus titer when compared to DMSO treatment. Because of cell toxicity at the initially tested concentration of some of the drugs, we repeated the assay with two lower concentrations, 10 μM and 2 μM, for 15 of the drugs. At 10 μM, 8 of 15 drugs demonstrated cell toxicity at 3 days post-treatment, and 3 drugs inhibited virus infection without noticeable cell toxicity. At the 2 μM concentration, 4 of the 15 drugs induced cell toxicity, and 2 drugs reduced the virus titer, producing no observable toxicity (Fig 1B and S3 Table). In summary, we identified four drugs (auranofin, Act-D, bortezomib, and gemcitabine) that induced strong mosquito cell toxicity, and two drugs (5-fluorouracil and MPA) that mediated significant inhibition of ZIKV infection of C6/36 cells without causing cell toxicity (Fig 1C). Next, the cytotoxicity of these 4 drugs to C6/36 cells was determined, and the IC_50_ of auranofin, Act-D, bortezomib, and gemcitabine was 254.0 ng, 405.8 ng, 4.5 ng and 50.2 ng, respectively (Fig 1D). The anti-ZIKV effect of 5-fluorouracil and MPA was also evaluated in C6/36 cells, and 5-fluorouracil significantly inhibited ZIKV infection at 2 μM and MPA inhibited ZIKV infection at 0.5 μM (Fig 1E).

### Mosquito adulticidal activities of anti-ZIKV drugs

**Fig 2:**
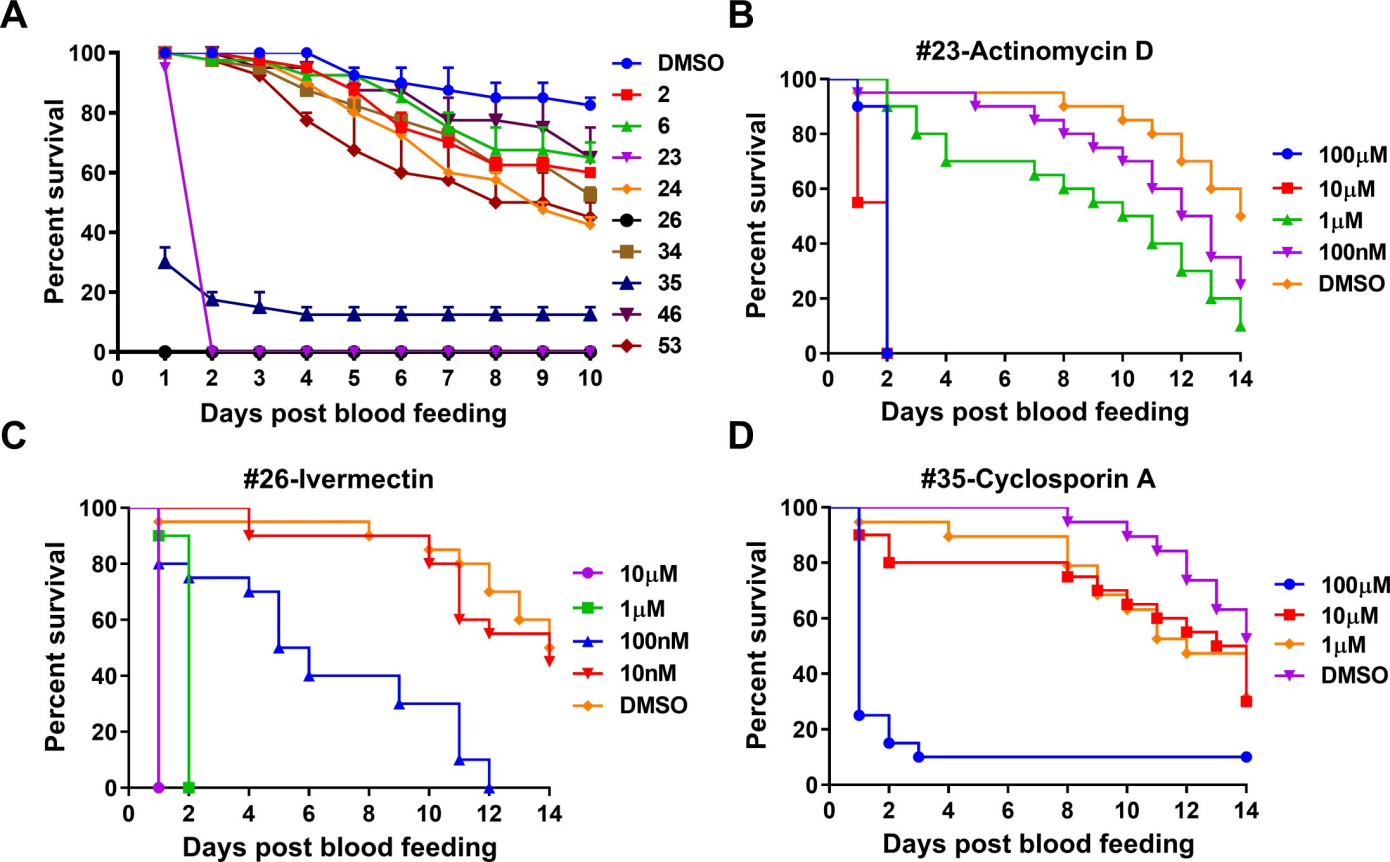
Adult mortality of *Aedes aegypti* fed on the nine selected anti-flaviviral drugs via a blood meal. **A**, Survival rates of adult females fed on the selected drugs. One-week old female mosquitoes were provided a blood feeding supplemented with each drug at a final concentration of 100 μM. The dead mosquitoes were counted each day for 10 days, and survival rates were calculated. Survival curves for adult female *Ae*. *aegytpi* fed with Act-D (**B**), ivermectin (**C**), or cyclosporin A (**D**) at various concentrations of each drug in the blood meal. The experiments were repeated twice, and one replicate was presented.

Since some drugs showed a strong toxicity to mosquito cells, we tested whether the 55 selected drugs influenced the viability of adult mosquitoes. Using an artificial membrane feeder, we orally fed each drug in combination with human blood, and monitored mosquito mortality daily up to 10 days. When adult female *Ae*. *aegypti* were fed 100 μM of each test drug in human blood, nine drugs induced mosquito mortality at 4 days and killed at least twice as many adults as the DMSO control at 10 days after exposure (Fig 2A and S4 Table). Three drugs showed a strong adulticidal activity, killing almost all the adult mosquitoes within 2 days (Fig 2A and S4 Table). When we serially 10-fold diluted these three drugs and tested them in adult females, we found that Act-D and cyclosporin A (CsA) exhibited a high mortality at 100 nM and 1 μM, respectively, while ivermectin induced the strongest mortality at 100 nM (Fig 2B–2D). Ivermectin is a known mosquitocidal for both *Aedes* and *Anopheles* species, whereas Act-D and CsA have not previously been reported to exert adulticidal activity.

### Anti-ZIKV drug activity in mosquitoes

**Fig 3:**
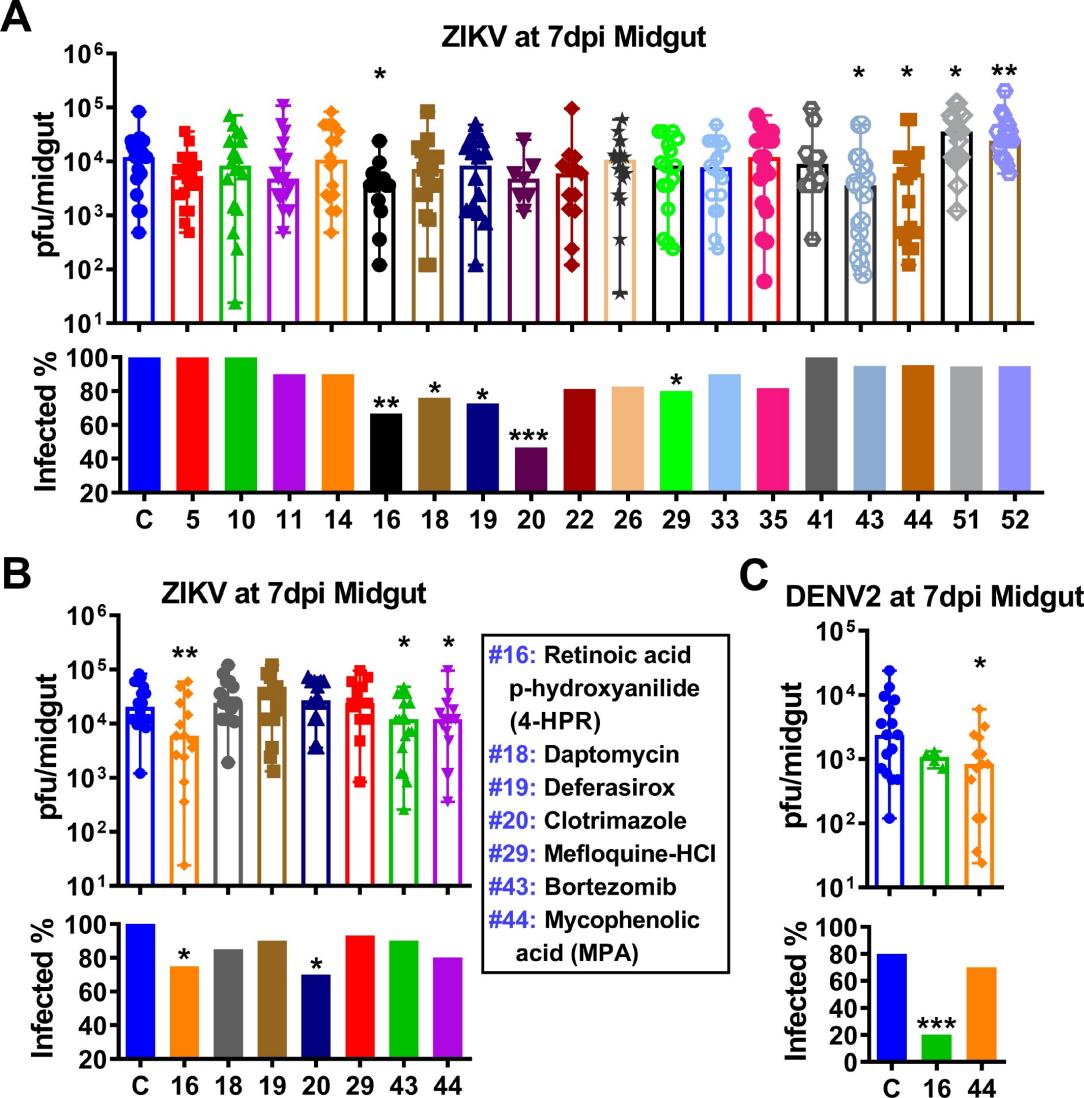
Anti-ZIKV infection of the 18 selected anti-flaviviral compounds tested in *Ae*. *aegypti*. **A and B,** ZIKV titers and infection prevalence of individual midgut of females fed with the selected drugs at 7 dpi, as determined by plaque assay in BHK cells. The final concentration of each drug in the ZIKV infectious blood meal was 100 μM, except #26 (10 nM) and #35 (1 μM). The ZIKV titers in the blood meal were 9×10^6^ plaque-forming units (pfu)/ml for **A**, and 1.3×10^7^ pfu/ml for **B**. **C,** DENV2 titers and the infection prevalence in individual midguts of mosquitoes fed with retinoic acid p-hydroxyanilide (4-HPR) or mycophenolic acid (MPA) with final concentration of 100 μM at 7 dpi, as determined by plaque assay in BHK cells. The DENV2 titer in the blood meal was 4×10^5^ pfu/ml. ZIKV and DENV2 were produced in C6/36 cells. Columns with error bars represent the median value with range of virus titer. *P*-values were calculated by a Mann-Whitney test for infection intensity or a Fisher's exact test for infection prevalence. *P<0.05, **P<0.01, ***P<0.001. One replicate for A, and at least two replicates for B and C.

Based on our C6/36 cell-based screening, 18 drugs that demonstrated cell toxicity or ZIKV inhibition were selected for testing in adult female *Ae*. *aegypti*. In a first assay, we fed one-week-old females with each drug at a final concentration of 100 μM in virus-containing blood, then measured midgut virus titers at 7 dpi by plaque assay. Because of the high toxicity of drugs #26 and #35, a final concentration of only 10 nM for #26 and 1 μM for #35 was used in the blood-virus mixture. The virus midgut infection intensity and prevalence were compared between each drug and the DMSO control. Three drugs (retinoic acid p-hydroxyanilide (4-HPR), bortezomib, and MPA) significantly reduced virus intensity, and five drugs (4-HPR, daptomycin, deferasirox, clotrimazole, and mefloquine-HCl) significantly reduced prevalence, whereas two drugs (#51: gemcitabine HCl and #52: clofazimine) actually significantly increased virus infection intensity (Fig 3A). It was particular noteworthy that 4-HPR dramatically reduced both virus infection intensity and prevalence. However, in the case of three drugs that reduced virus infection intensity in this first round of experiments (daptomycin, deferasirox, and mefloquine HCl), their ability to inhibit ZIKV could not be validated in a second round of virus challenge experiments with a higher ZIKV titer (Fig 3B); the higher viral load likely overcame the inhibitory action of these drugs. In addition, two drugs (4-HPR and MPA) also significantly reduced DENV2 infection intensity or prevalence in midguts in this second round (Fig 3C).

**Fig 4:**
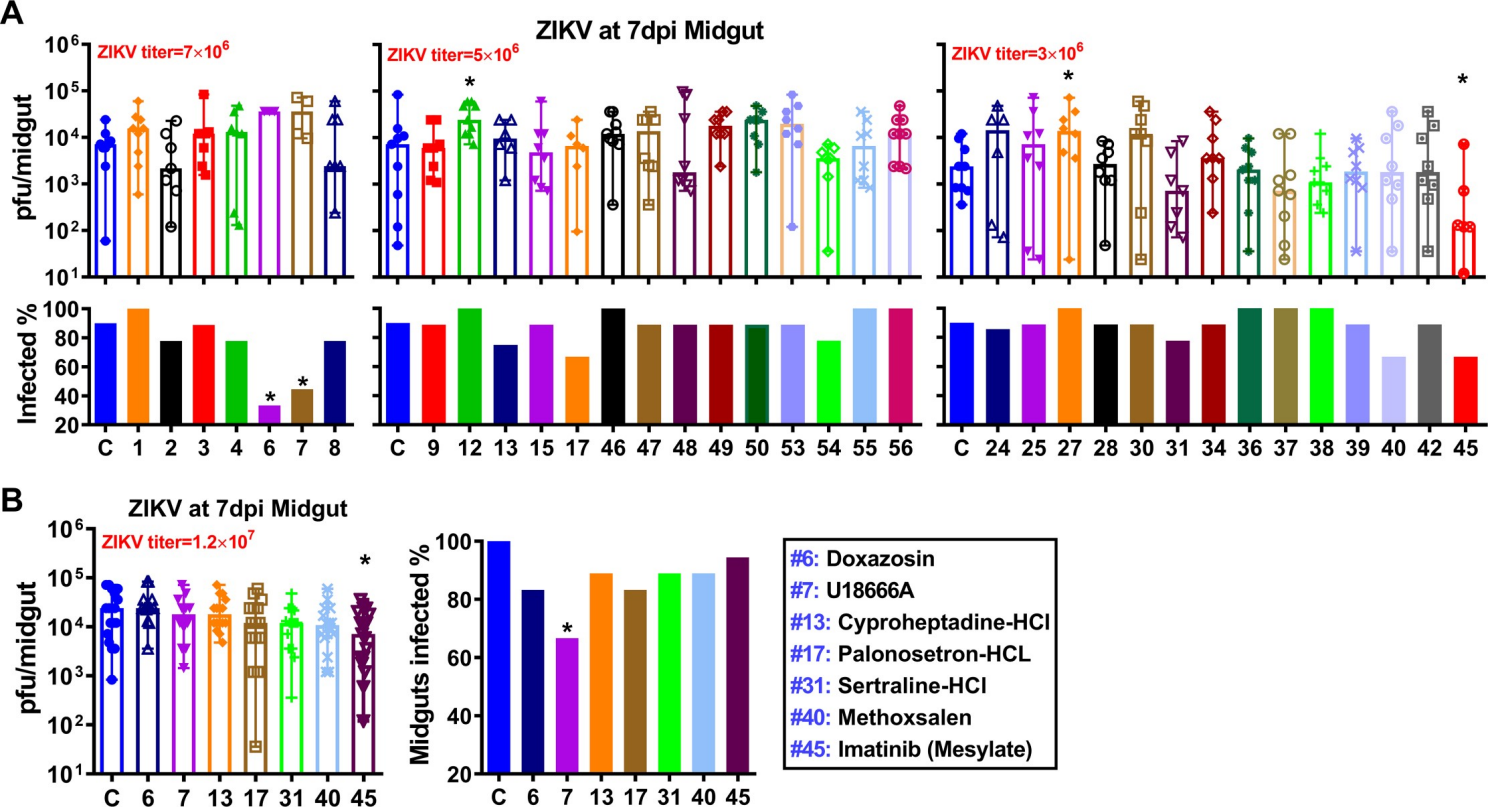
Anti-ZIKV activity of the rest of the anti-flaviviral drugs in *Ae*. *aegypti*. **A and B,** ZIKV titers and infection prevalence of individual midguts of females fed with each drug at 7 dpi, as determined by plaque assay in BHK cells. The final concentration of each drug in the ZIKV infectious blood meal was 100 μM, and the ZIKV titers in the blood meal are indicated in the figure. ZIKV were produced in C6/36 cells. Columns with error bars represent the median value with range of virus titer. *P*-values were calculated by a Mann-Whitney test for infection intensity or a Fisher's exact test for infection prevalence. *P<0.05, **P<0.01. One replicate for A, and at least two replicates for B.

**Fig 5:**
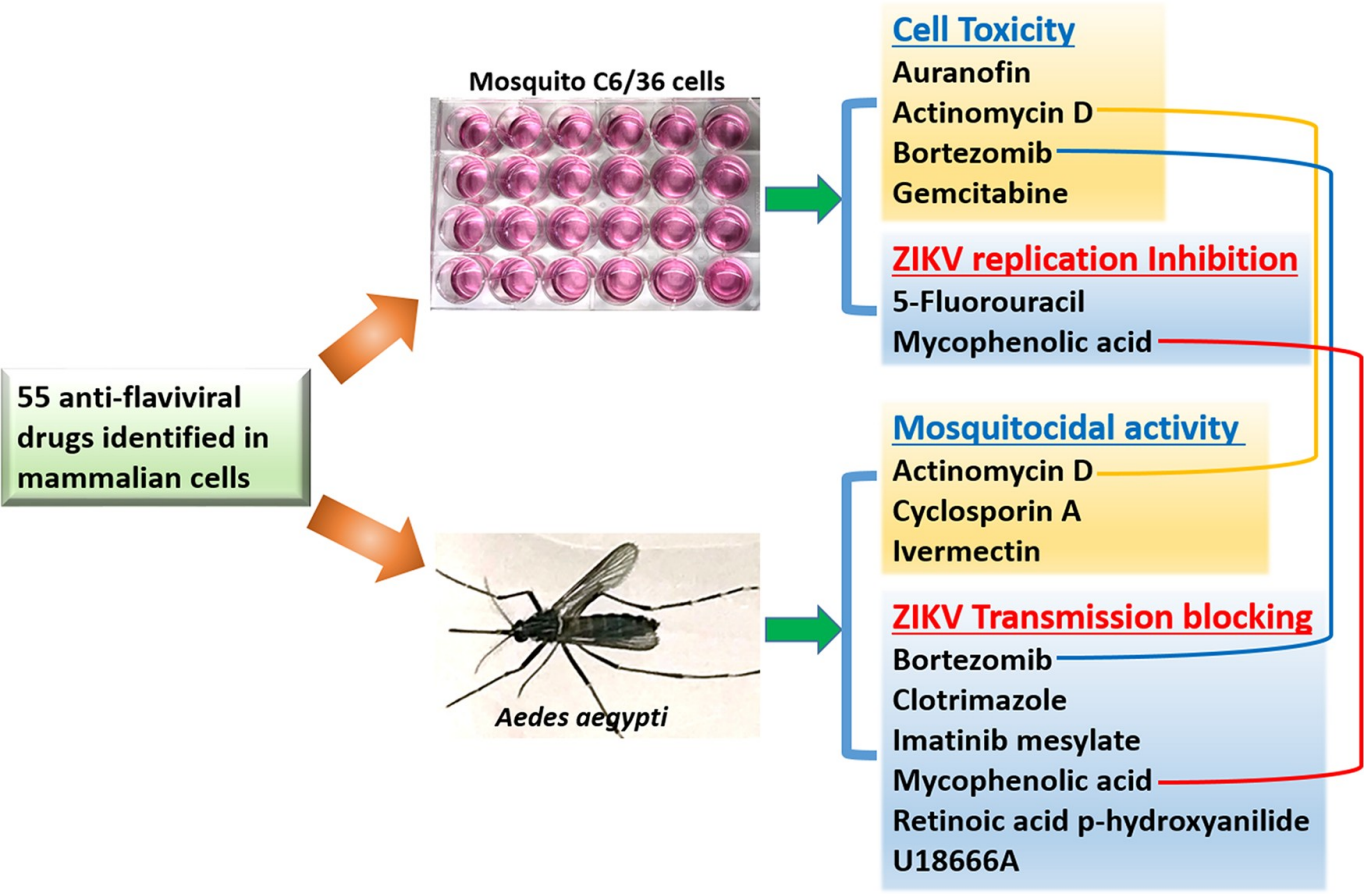
Compounds with mosquitocidal and anti-Zika activities in mosquito C6/36 cells or *Aedes aegytpi*.

Notably, two drugs (5-fluorouracil and clofazimine) inhibited ZIKV infection in mosquito cell lines but did not suppress ZIKV infection in mosquitoes, indicating differences in the cellular physiology and interactions with the virus *in vitro* and *in vivo* situations. Therefore, we tested all 55 drugs for anti-ZIKV activity in *Ae*. *aegypti*. In a pre-screen, nine females from each drug group were tested. One drug (imatinib mesylate) significantly decreased midgut infection intensity, and two drugs (doxazosin and U18666A decreased prevalence, whereas two drugs (#12: MG-132 and #27: pyrimethamine) significantly increased infection intensity (Fig 4A). However, we could not verify doxazosin as being able to reduce infection prevalence in the second virus challenge experiment using a higher ZIKV titer in a blood meal (Fig 4B). In summary, we identified six drugs (U18666A, 4-HPR, clotrimazole, bortezomib, MPA, and imatinib mesylate) (Fig 5) showing significant inhibition of ZIKV infection in *Ae*. *aegypti*.

Taken together, four and two drugs displayed a strong mosquito cell toxicity or significant inhibition of ZIKV replication, respectively, on the basis of C6/36 cells mortality and infection assays, three and six drugs possessed mosquitocidal or transmission blocking activity, respectively, as assayed by mosquito mortality and infection assays (Fig 5 and S5 Table). Additionally, MPA inhibits ZIKV infection in both C6/36 cells and adult female mosquitoes; Actinomycin D exerts mortality to both C6/36 cells and female adults, while Bortezomib exerts mortality to C6/36 cells and inhibits ZIKV infection in mosquito midguts.

## Discussion

Numerous small compounds have been described as exhibiting flavivirus-inhibiting activity in mammalian cells [17–19, 21]. However, only a few have been tested for anti-flaviviral activity in mosquito cells, and chemical inhibition of flavivirus infection in adult mosquitoes has only been reported by our recent study [14]. Here, we tested 55 FDA-approved drugs with known anti-flaviviral activity in mammalian cells for potential mosquitocidal and anti-ZIKV activity in mosquitoes and cells. Several drugs were found to exert adulticidal activity or suppress ZIKV infection in the midgut tissue, indicating that these drugs could be considered for further exploration as arboviral transmission-blocking agents. It is important to point out that some drugs (lovastatin[17, 18, 21], palonosetron-HCL [17, 18, 21] and 6-azauridine[18, 22]) have been shown to exhibit moderate anti-flaviviral activity in mammalian cells but did not affect ZIKV infection in either mosquito cells or adult females in our experiments, pointing to the existence of differences in host-virus interaction or drug-host interactions. This finding challenges the belief that the virus’ interaction with the vector and host entail mostly conserved molecular mechanisms and factors because of the small size of the viral genome.

Act-D was the only drug displaying toxicity to both mosquito cells and adult females. However, only a relatively high dosage of Act-D (10 μM in blood) exerted mosquitocidal activity within 2 days after feeding, suggesting this drug may not act as a highly potent mosquitocide. Act-D is a polypeptide antibiotic with activity against Gram-negative bacteria that has also been clinically used as an anticancer drug. The anticancer mechanism of Act-D involves its ability to inhibit DNA-dependent RNA polymerases [23]. Interestingly, Act-D also induces apoptosis in the lepidopteran insect cell line Sf21 (established from *Spodoptera frugiperda*) by inhibiting cellular RNA synthesis [24, 25]. It is likely that Act-D induces midgut cell apoptosis, thereby killing the insect.

Two drugs, ivermectin and CsA, also exhibited adulticidal activity. Ivermectin is a broad-spectrum antiparasitic agent, and previous studies have established it as a general mosquitocide (including *Aedes* and *Anopheles*) [26, 27]. A recent study has shown that the LC_50_ for ivermectin is about 2.5ng/ml in adult *Ae*. *aegypti* [26], which is similar to the lowest dosage used in our experiments. CsA is an immunosuppressive agent used in patients receiving organ transplants. CsA was first reported to exert mosquito larvicidal activity in 1988, killing *Culex pipiens autogenicus* L4 larvae upon exposure to CsA with a LC_50_ = ~0.6 μg/ml at 48 h [28]. Another study has shown that CsA is a metabolite forming the spore surface layer of *Tolypocladium tundrense* and *T*. *terricola*, which kill *Ae*. *aegypti* larvae by vacuolization and subsequent destruction of their midgut cell mitochondria [29]. In addition to its toxicity in mosquito larvae, CsA has also been reported to suppress the humoral immune response of the wax moth *Galleria mellonella* [30]. However, the adulticidal activity of CsA appeared to be weaker than the larvicidal activity, requiring 1 μM in the blood meal.

We report here that three drugs (5-fluorouracil and MPA) inhibited ZIKV infection of mosquito cells, and six drugs significantly suppressed ZIKV infection in mosquitoes, but only MPA showed a significant anti-flaviviral activity in both mosquito cells and adults. MPA is an inhibitor of inosine monophosphate dehydrogenase, which blocks the synthesis of xanthosine monophosphate. MPA inhibits flavivirus infection in mammalian cells by preventing the synthesis and accumulation of viral RNA [31, 32]. MPA also suppresses DENV2 infection in mosquito midguts and dissemination to salivary glands when exposed through a blood or sugar meal [14]. Our study revealed that MPA can also inhibit ZIKV infection in both mosquito cells and adults.

We also show that 4-HPR potently inhibits both ZIKV and DENV infection in mosquito midguts. 4-HPR is a retinoic acid (RA) derivative and a potential cancer-preventive agent that acts by inducing apoptosis in cancer cells [33], and has been extensively tested in humans [34, 35]. Recently, 4-HPR was also shown to be antiviral, since it inhibits DENV by limiting the accumulation of viral RNA in mammalian cells at the late stage of infection [36], as well as several other flaviviruses in mammalian cells [37]. It has also been shown that 4-HPR potently inhibits ZIKV infection in multiple mammalian and mosquito cell lines, and prophylactic delivery of the drug results in significant reductions in both serum viremia and brain viral burden in a murine ZIKV infection model [38, 39]. A significantly lower antiviral activity of 4-HPR has also been observed in C6/36 cells [39]. Accordingly, we did not detect a significant reduction in ZIKV titer in C6/36 cells treated with 4-HPR. However, 4-HPR significantly suppressed ZIKV infection and reduced DENV infection prevalence in mosquito midguts, suggesting that the inhibition likely occurs through viral host factors in mosquitoes. Further investigation of which host factors are involved in 4-HPR inhibition of ZIKV infection in mosquitoes is necessary to uncover the underlying mechanisms.

The FDA-approved 20S proteasome inhibitor bortezomib has been shown to inhibit infection of all four serotypes of DENV in primary monocytes, and bortezomib treatment of DENV-infected mice reduces viral load and signs of dengue pathology [40]. A recent study has also shown that bortezomib can suppress ZIKV infection in C6/36 cells in a dose-dependent manner, as well as reduce the viral load and signs of ZIKV pathology in treated mice [41]. Our C6/36 cell-based assay showed that ZIKV virus load was significantly reduced when cells were treated with 2 mM bortezomib, but treatment with the drug also affected cell viability, consistent with a study by Xin et al. that showed a concentration-dependent cytotoxicity in C6/36 cells (50% cytotoxic concentration (CC_50_) of >160 nM) [41]. Combined with our finding that bortezomib inhibited ZIKV infection in both mosquito cells and adult females, these results suggest that bortezomib is a good candidate to explore for blocking ZIKV transmission in mosquitoes and treatment of ZIKV infection in patients.

In addition to MPA, 4-HPR, and bortezomib, we also identified other three drugs (U18666A, clotrimazole, and imatinib mesylate) with activity against ZIKV infection in *Ae*. *aegypti*. U18666A is an amphipathic steroid that is widely used to block the intracellular trafficking of cholesterol by inhibiting oxidosqualene cyclase and desmosterol reductase [42]. U18666A has been shown to inhibit DENV entry and replication in mammalian cells by suppressing *de novo* sterol biosynthesis and retarding viral trafficking in the cholesterol-loaded late endosomes/lysosomes of host cells [43]. U18666A can also suppress infection and replication of the alphavirus Chikungunya virus (CHIKV) in human skin fibroblasts without any cytotoxic effects [44]. U18666A has not been tested to assess its effect on arbovirus infection in mosquitoes or insect cells. We found that U18666A significantly reduced ZIKV infection prevalence in mosquito midguts. Clotrimazole is a synthetic imidazole that acts against fungi by inhibiting the biosynthesis of sterols (ergosterol) of the fungal cell membrane, and it is widely used to treat fungal infections. Clotrimazole has been identified as suppressing ZIKV infection in mammalian cells [18]. Our study showed that clotrimazole also inhibits ZIKV infection in mosquito midguts. Its mechanism of inhibition of ZIKV infection has not been studied in mammalian cells or mosquitoes. Imatinib mesylate is an FDA-approved tyrosine kinase inhibitor that is used to treat several types of cancers. In addition to inhibiting ZIKV infection, imatinib also inhibits alphavirus (Sindbis virus) replication in mammalian cells [45].

The anti-flaviviral and mosquitocidal effects of the different compounds are most likely mediated by diverse mechanisms since their functions as therapeutic agents in humans are diverse. For example, MPA and CsA are used as immunosuppressants in kidney and liver transplant recipients [46, 47]; bortezomib, a proteinase inhibitor, is used to treat relapsed multiple myeloma and mantle cell lymphoma [48]; imatinib mesylate, a tyrosine kinase inhibitor, is used to treat chronic myelogenous leukemia (CML), gastrointestinal stromal tumors (GISTs) and a number of other malignancies [49]. The therapeutic dosage of drugs differ as does the drug concentration in patient’s blood or plasma. For example, serum concentration of imatinib mesylate ranges between 0.138 mg/ml and 2.816 mg/ml (median = 1.344 mg/ml) [50], which is higher than what we used for mosquito feeding (58.97 μg/ml); the patient serum level of MPA ranges from 0.45 to 6.5 mg/l (median = 2.1 mg/l) [51, 52], which is lower than that we fed mosquitoes on (32.04 μg/ml); the level of CsA in patient blood ranges from 212 to 1358 ng/ml [53], which is higher than that in blood meal can kill mosquitoes (120.26 ng/ml); the median concentration of Actinomycin D in patient plasma ranges from 24.4 to 128 μg/l between 5 and 15 min post administration [54], which is similar to that in blood meal can kill mosquitoes (125.5 ng/l). It is standard practice to use uniform concentrations when screening multiple compounds for bioactivity such as anti-viral action [17, 55]. Since we did not observe complete virus-blocking (infection intensity of zero) for any of the drugs, even at concentrations higher than patient serum levels, we did not pursue testing of lower concentrations. Nevertheless, the mosquitocidal or virus-blocking concentration is irrelevant when considering the design of a control strategy based on artificial toxic sugar bait (ATSB) delivery of compounds to mosquitoes[16, 56].

In conclusion, we have screened 55 FDA-approved drugs with known anti-flaviviral activity in mammalian and/or mosquito cells. Based on C6/36 cell assays, four drugs (auranofin, Act-D, bortezomib, and gemcitabine) were identified as exerting moderate toxicity to mosquito cells, and two drugs (5-fluorouracil and MPA) were identified as significantly reducing ZIKV infection. Our mosquito mortality assays revealed that nine drugs had mosquitocidal activity, and three of these (Act-D, CsA, and ivermectin) exhibited a moderate adulticidal activity and should therefore be further explored for utility in mosquito control. Finally, six drugs (U18666A, 4-HPR, clotrimazole, bortezomib, MPA, and imatinib mesylate) demonstrated anti-ZIKV activity in mosquito midguts, and three of them (MPA, 4-HPR, and bortezomib) showed particularly potent ZIKV-blocking ability, rendering them interesting candidates for the development of transmission-blocking strategies.

## Materials and methods

### Ethics statement

This study was carried out in accordance with the recommendations in the Guide for the Care and Use of Laboratory Animals of the National Institutes of Health, the Animal Care and Use Committee (ACUC) of the Johns Hopkins University, and the institutional Ethics Committee (permit number: M006H300). The IACUC committee approved the protocol. Mice were only used for mosquito rearing. Commercial, anonymous human blood was used for virus infection assays in mosquitoes, and informed consent was therefore not applicable.

### Chemicals and viruses

The chemicals used in this study were purchased from Sigma or MedChemExpress (MCE). ZIKV (Cambodia, FSS13025) and DENV serotype 2 (New Guinea C strain, DENV2) were used.

### Mosquito rearing and cell culture

*Ae*. *aegypti* (Rockefeller) larvae were reared with fish food, and adults were maintained on a 10% sucrose solution at 27°C and 85% humidity with a 12-h light/dark cycle. The mice were used for blood feeding and colony maintenance. The C6/36 (*Ae*. *albopictus*) cell line that was used for virus propagation was grown in minimal essential medium (MEM, Gibco, Carlsbad, CA, USA) with 10% heat inactivated FBS, 1% L-glutamine, 1% penicillin-streptomycin, and 1% non-essential amino acids at 32°C with 5% CO_2_. The baby hamster kidney BHK-21 and Vero cell lines that were used for plaque assays were maintained on Dulbecco's modified Eagle's medium (DMEM, Gibco, Carlsbad, CA, USA) supplemented with 10% FBS, 1% L-glutamine, 1% penicillin-streptomycin, and 5 μg/ml plasmocin (Invitrogen, Carlsbad, CA) at 37°C with 5% CO_2_.

### ZIKV infections and drugs screening assay with C6/36 cells

Day 1: C6/36 cells were cultured in 24-well plates, then used for ZIKV infection when the cells were 80% confluent. Day 2: The cells were pretreated with drugs or controls. Drugs were diluted to a final concentration of 20 μM (or 10 μM or 2 μM) in complete MEM medium and added to the cells, which were incubated at room temperature in the rocking machine for 15 min, then incubated for 1 h in an incubator at 32°C with 5% CO_2_. Approximately 1 h after the addition of a test compound, ZIKV was added to each well at an MOI of 0.5. Infected cells were incubated at 32°C with 5% CO_2_. Each drug treatment had at least three replicates. Day 3–5: The cells were observed while growing, and the floating and highly granulated deformed cells were noted under the microscope and recorded each day. Day 5: If the mock-infected cells showed signs of infection, 200 μl of conditioned cell culture medium was harvested from each well and kept at -80°C for virus tittering. To measure IC_50_ of the selected drugs, C6/36 cells were plated at densities of 1 × 10^4^ cells/well in 96-well plates. The cells were incubated overnight and then treated with the compounds at a final concentration of 10 μM, 2 μM, 0.5 μM, 0.1 μM, 20 nM, 5 nM, 1 nM, 0.2 nM and 0.05 nM, respectively. C6/36 cells were incubated for another 96 h. Cell viability was measured using the CellTiter 96 Aqueous One Solution Reagent (Promega, Madison, WI, USA) and a Spectramax M2 plate reader (Molecular Devices, Sunnyvale, CA, USA) following manufacturer's protocol. Results from drugs treated cells were compared to results from DMSO controls. The experiments were performed in triplicate. IC_50_ of each drug was determined using nonlinear regression with GraphPad Prism.

### Virus infection of mosquitoes

ZIKV or DENV2 was propagated in C6/36 cells in T25 flasks and infected with ZIKV at an MOI of 0.5 or DENV2 at an MOI of 0.1 using MEM with 10% FBS, 1% L-glutamine, 1% penicillin-streptomycin, and 1% non-essential amino acids at 32°C with 5% CO_2_. After 5–6 days of incubation at 28°C, infected cell culture medium was mixed with an equal volume of commercial human blood supplemented with 10% human serum containing 10 mM ATP and a final concentration of 100 μM of the test drug. One week-old females were fed for 30 min with the mixture at 37°C using a single glass feeder per cup. Fully engorged females were selected and maintained on 10% sucrose at 27°C and 85% humidity with a 12-h light/dark cycle. Midguts were dissected from individual females at 7 dpi and kept it at -80°C.

### Sample processing and plaque assay

Samples harvested from cell culture were diluted 1:1000 with DMEM, and 10 μl were used for a plaque assay. Infected mosquito midguts were homogenized in DMEM with a Bullet Blender (Next Advance Inc., Averill Park, NY, USA) with glass beads, centrifuged at 10,000g for 5 min at 4°C, and the supernatants were harvested for plaque assay. DENV2 was tittered with BHK 21 cells, and ZIKV for C36/36 cell-screening was tittered with Vero cells and for mosquito infection assay with BHK cells. In brief, infected medium or midgut samples were serially 10-fold diluted and inoculated into cells seeded and grown to 80% confluence in 24-well plates. Plates were rocked for 15 min at RT, followed by incubation at 37°C with 5% CO_2_ for 45 min. Subsequently, 1 ml of DMEM containing 2% FBS, 0.8% methylcellulose and 1% penicillin-streptomycin were added to each well, and the plates were incubated for 5 days at 37°C with 5% CO_2_. The plates were then fixed and visualized with methanol/acetone mixture (1:1 volume) and 1% crystal violet mixture for 30 min at RT, and the plaque-forming units were counted.

### Mosquito mortality assay

Thirty one-week-old female mosquitoes were transferred to a paper cup the day before blood feeding. Mosquitoes were fed with human blood containing the tested drug on a glass feeder. Twenty fully engorged mosquitoes per cup were retained for a survival study. For the initial screening, a final concentration of 100 μM was tested for each drug. For those chemicals showing high mortality, a 10-fold serial dilution was used for the next round of screening. Dead mosquitoes were recorded and removed every day for 10 days or 14 days. Each chemical feeding had at least three replicates. Survival curves were generated using GraphPad Prism.

### Statistical analyses

Statistical analysis was performed using GraphPad Prism. Differences in virus intensity and prevalence of infection in midguts between chemical treatment and the control were compared using the non-parametric Mann-Whitney U-test or Fisher's exact test, respectively. In the experiments screening C6/36 cells, data from each sample were analyzed using Student’s *t*-test. All tests were considered significant at *P* < 0.05.

## Supporting information

S1 Table The selected 55 drugs with anti-flavivirus infection in mammalian cells. (XLSX)

S2 Table Screening anti-flavivirus drugs for anti-ZIKV infection and cell toxicity to mosquito cells at 100 μM in C6/36 cells. (XLSX)

S3 Table Mosquito cell screening of anti-flavivirus drugs at 10 μM and 2 μM. (XLSX)

S4 Table Mosquito mortality assay of anti-flavivirus drugs at 100 μM. (XLSX)

S5 Table Summarize the drugs with mosquitocidal and anti-Zika activities. (XLSX)
